# Sequential disruption of SPLASH-identified vRNA–vRNA interactions challenges their role in influenza A virus genome packaging

**DOI:** 10.1093/nar/gkad442

**Published:** 2023-05-24

**Authors:** Celia Jakob, Gabriel L Lovate, Daniel Desirò, Lara Gießler, Redmond P Smyth, Roland Marquet, Kevin Lamkiewicz, Manja Marz, Martin Schwemmle, Hardin Bolte

**Affiliations:** Institute of Virology, Medical Center – University of Freiburg, Freiburg, Germany; Faculty of Medicine, University of Freiburg, Freiburg, Germany; RNA Bioinformatics and High-Throughput Analysis, Faculty of Mathematics and Computer Science, Friedrich Schiller University Jena, Germany; Department of Biochemistry, University of Cambridge, CambridgeCB2 1QW, UK; Institute of Virology, Medical Center – University of Freiburg, Freiburg, Germany; Helmholtz Institute for RNA-based Infection Research, Helmholtz Centre for Infection Research, Würzburg, Germany; Julius-Maximilians-Universität Würzburg, Faculty of Medicine, Würzburg, Germany; Architecture et Réactivité de l’ARN, Université de Strasbourg, CNRS, IBMC, Strasbourg, France; RNA Bioinformatics and High-Throughput Analysis, Faculty of Mathematics and Computer Science, Friedrich Schiller University Jena, Germany; German Center for Integrative Biodiversity Research (iDiv), Halle-Jena-Leipzig, Germany; European Virus Bioinformatics Center (EVBC), Jena, Germany; RNA Bioinformatics and High-Throughput Analysis, Faculty of Mathematics and Computer Science, Friedrich Schiller University Jena, Germany; German Center for Integrative Biodiversity Research (iDiv), Halle-Jena-Leipzig, Germany; European Virus Bioinformatics Center (EVBC), Jena, Germany; FLI Leibniz Institute for Age Research, Jena, Germany; Institute of Virology, Medical Center – University of Freiburg, Freiburg, Germany; Faculty of Medicine, University of Freiburg, Freiburg, Germany; Institute of Virology, Medical Center – University of Freiburg, Freiburg, Germany; Faculty of Medicine, University of Freiburg, Freiburg, Germany

## Abstract

A fundamental step in the influenza A virus (IAV) replication cycle is the coordinated packaging of eight distinct genomic RNA segments (i.e. vRNAs) into a viral particle. Although this process is thought to be controlled by specific vRNA–vRNA interactions between the genome segments, few functional interactions have been validated. Recently, a large number of potentially functional vRNA–vRNA interactions have been detected in purified virions using the RNA interactome capture method SPLASH. However, their functional significance in coordinated genome packaging remains largely unclear. Here, we show by systematic mutational analysis that mutant A/SC35M (H7N7) viruses lacking several prominent SPLASH-identified vRNA–vRNA interactions involving the HA segment package the eight genome segments as efficiently as the wild-type virus. We therefore propose that the vRNA–vRNA interactions identified by SPLASH in IAV particles are not necessarily critical for the genome packaging process, leaving the underlying molecular mechanism elusive.

## Graphical Abstract

**Graphical Abstract:**
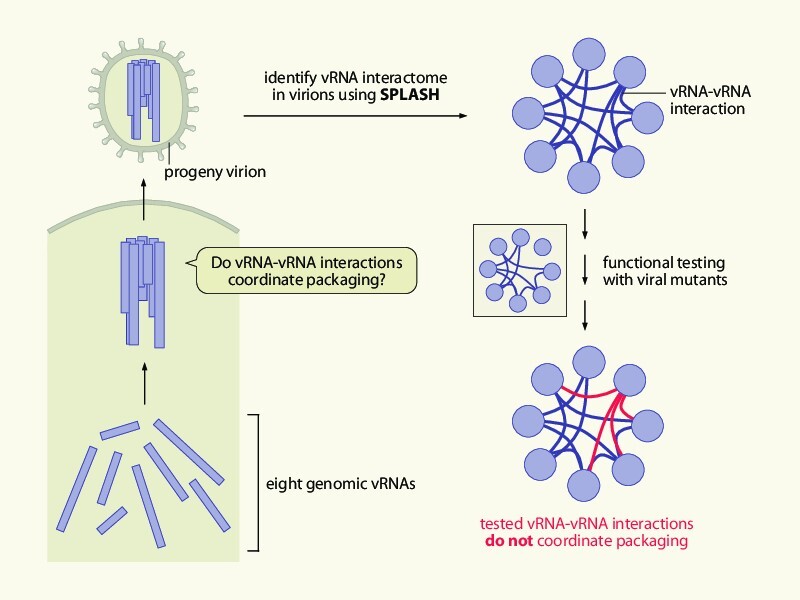

## INTRODUCTION

Zoonotic influenza A viruses (IAVs) from avian and mammalian species can cause severe disease in humans. Typically, such spillover events are rare and limited to a few infected individuals due to the poor adaptation of zoonotic IAVs to humans (1). Occasionally, however, zoonotic IAVs emerge that replicate efficiently in humans and cause pandemics (2,3). These pandemic IAVs can arise through genetic reassortment when different IAV strains exchange parts of their segmented genomes in co-infected cells (4). These novel genome constellations facilitate viral adaptation and lead to pandemics with high morbidity and mortality, as occurred in 1957, 1968 and 2009 (5–7). Genetic reassortment is thought to be controlled by a coordinated genome packaging process in which eight unique genome segments are selectively incorporated into a viral particle (8,9). However, despite its central role in the emergence of pandemic IAVs, the molecular mechanisms underlying the genome packaging process are poorly understood.

All eight viral RNAs (vRNAs) are organized into a central open reading frame (ORF) flanked by short non-coding regions (NCRs) required for segment replication and transcription (10–13). The vRNAs are bound by multiple copies of the viral nucleoprotein (NP) and at the genome termini by a heterotrimeric viral polymerase, which together form eight viral ribonucleoproteins (vRNPs) (14–17). Terminal packaging sequences (TPS), typically composed of the NCRs and adjacent terminal portions of the ORF, have been identified in all vRNAs (18–36). TPS are critical for coordinated packaging of all eight genome segments, as their deletion or synonymous mutation is associated with decreased viral infectivity, increased production of noninfectious virions, and impaired packaging of one or more vRNAs.

Many studies have postulated the existence of a vRNA–vRNA interaction network that controls the assembly of a supramolecular complex consisting of eight different vRNPs (18,31,34,37–41). This has led to the suggestion that TPS form specific vRNA–vRNA interactions between the vRNPs, but despite some supporting evidence (32,42), specific base pairings of the TPS that are critical for genome packaging have not yet been revealed. In contrast, two functional interactions involving vRNA regions beyond the TPS have been identified (38,40,43). However, apart from these two validated interactions, the architecture of the genome packaging interaction network remains unresolved.

The recent development of techniques combining RNA–RNA cross-linking with next-generation sequencing has enabled high-throughput mapping of vRNA–vRNA contacts in viral particles (37,38,44). Using sequencing of psoralen cross-linked, ligated, and selected hybrids (SPLASH) (45,46), Dadonaite *et al.* demonstrated the existence of complex and redundant vRNA–vRNA interaction networks in purified virions of several IAV strains (38). Importantly, a previously proposed (43) vRNA–vRNA interaction between the PB1 and NA segments of H3N2 viruses was confirmed by SPLASH, and its functional importance in IAV genome packaging was validated by mutational analysis (38). However, it remains unclear whether SPLASH is able to detect functional vRNA–vRNA interactions on a large scale across IAV strains, as a systematic evaluation of the detected networks is still lacking.

Here, we systematically evaluated the functional significance of SPLASH-identified vRNA–vRNA interactions in the genome packaging process of an H7N7 virus by generating viral mutants lacking several prominent HA segment interactions. In addition, we tested the relevance of SPLASH-identified vRNA–vRNA interactions involving TPS of the HA and NA segments.

## MATERIALS AND METHODS

### Cell culture

MDCK-II cells (ATCC; CRL-2936) and HEK 293T cells (ATCC; CRL-3216) were grown at 37°C and 5% CO_2_, in Dulbecco's modified Eagle's medium (DMEM; Gibco 41966-029) supplemented with 10% fetal calf serum (FCS, anprotec; AC-SM-0190) and 1% penicillin–streptomycin (pen/strep; Gibco 15140–122).

### Plasmids

All plasmids used in this study are listed in Supplementary Table S1. The pHW2000 plasmids encoding genome segments and proteins of wild-type A/Seal/Massachusetts/1/1980 (H7N7) (designated SC35M) (47) and rPB1_TPS_ (34) have been described previously. The pHW2000 plasmids used to generate rHA_4x_, rHA_4x_ + 4x, rHA_TPS_, rM_mut_, rNA_TPS_, rHA_mut_, rPB1_TPS+mut_ or combinations thereof, were generated by site-specific mutagenesis using the primers listed in Supplementary Table S1. The pHW2000 plasmids encoding vRNAs and proteins of A/Puerto Rico/8/34 (H1N1) (designated PR8), A/Wyoming/3/03 (H3N2) PB1 (Wy-PB1), Wy-NA and Wy-NA_Ud-sub_ have been described previously and were a kind gift from Brad Gilbertson (43).

### Generation of recombinant influenza A viruses

Recombinant SC35M and chimeric PR8 viruses were generated using an eight-plasmid reverse-genetics system (48) (Supplementary Table S1). Subconfluent HEK 293T cells in 6-well plates were transfected with 500 ng of each pHW2000 plasmid using Lipofectamine 2000 (Thermo Fisher Scientific; 11668019) according to the manufacturer's protocol. Six hours (h) after transfection, the Opti-MEM medium was replaced with DMEM containing 0.2% bovine serum albumin (BSA; AppliChem A1391) and 1% pen/strep. After 48–72 h, the viral supernatant was collected and purified by plaque assay on MDCK-II cells. Individual plaques were picked and amplified on MDCK-II cells to generate clonal SC35M virus stocks. For chimeric rPR8:Wy-PB1/NA and rPR8:Wy-PB1/NA_Ud-sub_ viruses, the rescue supernatant was first amplified on MDCK-II cells in the presence of 1 μl per milliliter (ml) TPCK-treated trypsin (Thermo Fisher Scientific; 20233) and then plaque-purified. Mutations in the viral genome were confirmed by Sanger sequencing. Briefly, 200 μl virus supernatant was mixed with 600 μl Trizol reagent (Ambion; 15596026) and purified using the Direct-zol RNA Miniprep Kit (Zymo Research; R2050). 5 μl of RNA was reverse-transcribed and amplified using segment-specific primers (Supplementary Table S1) and the OneStep RT-PCR Kit (Qiagen; 210212). Purified DNA was sequenced by Eurofins Genomics or Genewiz.

### Multicycle virus replication kinetics

Confluent MDCK-II cells in 6-well plates were washed with PBS and infected with wild-type or mutant SC35M virus at a multiplicity of infection (MOI) of 0.001 plaque-forming units (PFU) per cell in DMEM containing 0.2% BSA and 1% pen/strep. For chimeric PR8 viruses, the infection medium contained 1 μl per ml of TPCK-treated trypsin. Supernatants were collected at 24 h post infection (hpi) and PFU titers were determined by plaque assay on MDCK-II cells.

### Measurements of relative HAU-to-PFU ratios

Confluent MDCK-II cells in 6-well plates were washed with PBS and infected with wild-type or mutant SC35M virus at an MOI of 0.001 PFU per cell in DMEM containing 0.2% BSA and 1% pen/strep. Supernatants were harvested at 24 or 36 hpi. PFU titers were determined by plaque assay on MDCK-II cells. Hemagglutination titers were determined by HA assay as described previously (49). Briefly, chicken erythrocytes (Labor Merk; E-200) were diluted to 0.75% (v/v) in PBS and added to a 1:2 serial dilution of viral supernatant in a 96-round well plate. After 30 to 60 minutes (min) of incubation at room temperature, individual wells were monitored for hemagglutination. The HA titer (in hemagglutination units (HAU) per 50 μl) was the lowest virus dilution that produced hemagglutination. The relative log_2_ HAU to PFU ratio of the mutant (mut) compared to the wild-type (WT) virus was calculated as follows:


$$\begin{eqnarray*} && {\rm{lo}}{{\rm{g}}}_2{\rm{HAU}}\,{\rm{to}}\,{\rm{PFU}}\,{\rm{ratio}} = \left( {{\rm{HA}}{{\rm{U}}}_{{\rm{mut}}} - {\rm{HA}}{{\rm{U}}}_{{\rm{WT}}}} \right)\nonumber\\ && \quad - \left( {{\rm{lo}}{{\rm{g}}}_2{\rm{PF}}{{\rm{U}}}_{{\rm{mut}}} - {\rm{lo}}{{\rm{g}}}_2{\rm{PF}}{{\rm{U}}}_{{\rm{WT}}}} \right).\end{eqnarray*}$$

### Relative quantification of packaged vRNAs by RT-qPCR

Confluent MDCK-II cells in 6-well plates were washed with PBS and infected with wild-type and mutant SC35M viruses at an MOI of 0.001 PFU per cell in DMEM containing 0.2% BSA and 1% pen/strep. Supernatants were collected at 24 or 36 hpi. For RNA isolation, 200 μl of virus supernatant was mixed with 600 μl Trizol and purified using the Direct-zol RNA Miniprep kit. 4 μl of RNA was reverse transcribed using random hexamer primers and the RevertAid First Strand cDNA Synthesis Kit (Thermo Fisher Scientific; K1621) according to the manufacturer's protocol. 1:100 dilutions of the cDNA products and 400 nM of segment-specific primers (Supplementary Table S1) were used for quantitative PCR (qPCR) using the SensiFast SYBR Hi-ROX kit (Bioline; BIO-92020) according to the manufacturer's instructions. Reactions were performed in technical triplicate using the Quant Studio 5 Real-Time PCR System (Thermo Fisher Scientific; A34322). After excluding a packaging defect of the PB2 segment, vRNA levels in the mutants were normalized to those of the wild type and then to the relative PB2 vRNA levels as follows:


$$\begin{eqnarray*} &&{\rm{\Delta \Delta CT}} = \left( {{\rm{C}}{{\rm{T}}}_{{\rm{WT\_vRNA\_x}}} - {\rm{C}}{{\rm{T}}}_{{\rm{mut\_vRNA\_x}}}} \right)\nonumber\\ && \quad - \left( {{\rm{C}}{{\rm{T}}}_{{\rm{WT\_vRNA\_PB2}}} - {\rm{C}}{{\rm{T}}}_{{\rm{mut\_vRNA\_PB2}}}} \right)\end{eqnarray*}$$

### SPLASH

SPLASH was performed as previously described (38,45,46) with minor modifications. Briefly, confluent MDCK-II cells in T75 flasks were washed with PBS and infected with wild-type or mutant SC35M virus at an MOI of 0.001 PFU per cell in DMEM containing 0.2% BSA and 1% pen/strep. For chimeric PR8 viruses, the infection medium contained 1 μl per ml of TPCK-treated trypsin. Supernatants were collected at 24 to 36 hpi, cleared of debris by centrifugation (30 min, 2000 rpm, Eppendorf rotor S-4–104, 4°C), and concentrated by ultracentrifugation (1.5 h, 25 000 rpm, Beckman Coulter rotor SW32 Ti, 4°C) through a 30% (w/v) sucrose cushion. Purified virus was added to a 24-well plate and treated with 200 μM EZ-Link Psoralen-PEG3-Biotin (Thermo Fisher Scientific; 29986) and 0.01% digitonin (Sigma-Aldrich; D141) for 5 min at 37°C. The samples were cross-linked with UV light at 365 nm for 20 min on ice in a UVP CL-1000L cross-linker (Analytik Jena). Samples were digested with 0.25 mg/ml of proteinase K (Thermo Fisher Scientific; E00491) in proteinase K buffer (0.5% SDS, 0.1 M Tris–HCl pH 7.5, 50 mM NaCl, 10 mM EDTA) for 20 min at 37°C with shaking. Samples, were solubilized in Trizol LS reagent (Ambion; 10296028) and chloroform. RNA was extracted from the aqueous phase using the RNA Clean & Concentrator-5 Kit (Zymo Research; R1013). Purified RNA was fragmented using the NEB Next Magnesium RNA Fragmentation Module (NEB; E6150S) at 94°C for 4 min. RNA fragments were purified using the RNA Clean & Concentrator-5 Kit, and enriched for biotinylated RNA by binding to Hydrophilic Streptavidin Magnetic Beads (NEB; S1421S). Briefly, 100 μl of bead suspension was washed twice with lysis buffer (50 mM Tris–HCl pH 7, 10 mM EDTA, 1% SDS). The beads were resuspended in 60 μl lysis buffer supplemented with 3.5 μl per ml of Superase-In (Invitrogen; AM2694), and the purified RNA was added to the beads along with 600 μl supplemented lysis buffer and 1200 μl hybridization buffer (750 mM NaCl, 1% SDS, 50 mM Tris–HCl pH 7, 1 mM EDTA, 15% formamide). After shaking for 30 min at 37°C, the beads were washed five times with wash buffer (10% SSC (Sigma-Aldrich; S6639-1L), 0.5% SDS) by shaking for 5 min at 37°C. To remove 3′-cyclic phosphate groups, the beads were washed twice with cold PNK-T buffer (70 mM Tris–HCl pH 7.7, 10 mM MgCl2, 5 mM DTT, 0.1% Tween-20) for 5 min on a rotating wheel at 4°C and then resuspended in 150 μl of T4 PNK mix (122.25 μl H2O, 1.5 μl 10% Tween-20, 15 μl 10x T4 PNK buffer, 3.75 μl Superase-In, 7.5 μl T4 PNK (NEB; M0201L)). After 4 h of shaking at 37°C, 37.5 μl of phosphorylation mix (3.75 μl 10x T4 PNK buffer, 9.4 μl T4 PNK, 18.8 μl 10 mM ATP, 5.6 μl H2O) were added to the samples and incubated for 60 min at 37°C with shaking. The reaction was stopped by adding 400 μl of cold RnlT buffer (50 mM Tris–HCl pH 7.5, 10 mM MgCl_2_, 1 mM DTT, 0.1% Tween-20). The beads were washed twice with cold RnlT buffer and incubated for 5 min at 4°C on a rotating wheel. The beads were resuspended in 100 μl ligation mix (10 μl 10x T4 RNA ligase buffer, 1 μl 100 mM ATP, 2.5 μl Superase-In, 8.3 μl T4 Rnl1 (NEB; M0437M), 1 μl 10% Tween-20, 77.2 μl H_2_O) and shaken for 16 h at 16°C. The beads were washed twice with wash buffer and the RNA was eluted by resuspending the beads in 400 μl Trizol. The RNA was purified with the RNA Clean & Concentrator-5 Kit and reverse cross-linked with UV light at 254 nm for 5 min on ice. The RNA was concentrated with the RNA Clean & Concentrator-5 Kit and subjected to library preparation using the SMARTer smRNA-Seq Kit (Takara; 635029) according to manufacturer's protocol. RNA size selection was performed by binding to Ampure XP beads (Beckman Coulter; A63880). Final libraries were pooled and sequenced on an Illumina NextSeq 550 instrument (1 × 150–160 cycles) using the NextSeq 500/550 High Output Kit v2.5 (Illumina; 20024907).

### CAPTIVE

Confluent MDCK-II cells in 15-cm dishes were washed with PBS and inoculated on ice with SC35M virus at an MOI of 3 PFU per cell in DMEM containing 0.2% BSA and 1% pen/strep. After 45 min on ice, the cells were incubated at 37°C for another 45 min. The viral supernatant was removed and the cells were washed five times with pre-warmed PBS. Infected cells were further cultured in the presence of 100 μM zanamivir (Sigma-Aldrich; SML0492) for 13 h at 37°C. To study the RNA interactome in newly formed cell-attached virus particles, infected cells were washed with warm NT buffer (0.1 M NaCl, 20 mM Tris–HCl pH 8) and treated with 720 μM psoralen-TEG-azide (Berry & Associates; PS 5030) dissolved in warm NT buffer for 10 min at 37°C. Cells were cooled on ice for 5 min, followed by RNA–RNA cross-linking with UV light at 365 nm for 10 min. Virions were released from the cells by treatment with 25 milliunits per ml of *Vibrio cholera* sialidase (Merck; N7885) in NTC buffer (0.1 M NaCl, 20 mM Tris–HCl pH 8 and 5 mM CaCl_2_) at 37°C for 1.5 h. Released virions were cleared of debris by centrifugation (30 min, 2000 rpm, Eppendorf rotor S-4–104, 4°C) and concentrated by ultracentrifugation (1.5 h, 25 000 rpm, Beckman Coulter rotor SW32 Ti, 4°C) through a 30% (w/v) sucrose cushion. To study the vRNA interactome in released virus particles, cell-attached virions were first released by sialidase treatment, followed by RNA–RNA cross-linking with 200 μM of psoralen-TEG-azide. To study the vRNA interactome in purified virions, RNA–RNA cross-linking with 200 μM of psoralen-TEG-azide was performed after sialidase treatment and ultracentrifugation. Purified virions were lysed in DNA/RNA Shield (Zymo Research; R1100) and digested with 0.4 mg per ml of proteinase K for 60 min at room temperature. Viral RNA was purified using the RNA Clean & Concentrator-5 Kit and further processed as described in the ’SPLASH’ section with the following modifications: Briefly, the fragmented RNA was biotinylated overnight with 50 μM sDIBO-biotin alkyne (Thermo Fisher Scientific; C20023) in PBS at room temperature. The biotinylated RNA was then enriched using Dynabeads MyOne Streptavidin C1 (Thermo Fisher Scientific; 65001).

### Bioinformatical analyses of SPLASH datasets using RNAswarm

The workflow described below is a mixture of automated steps and manual curation. The script package used is called RNAswarm and can be found in our GitHub repository (https://github.com/gabriellovate/RNAswarm/releases/tag/v0.1.0). We first pre-processed the raw Illumina SPLASH data by trimming adapters and filtering for quality using fastp (50) (version 0.23.2, default parameters). The corresponding fastqc files (51) (version 0.11.8, default parameters) before and after trimming are available in our OSF repository (https://osf.io/berj7/). To detect chimeric reads resulting from cross-linking of intra- and intersegmental RNA–RNA interactions, we mapped the pre-processed sequencing data to all eight segments of the corresponding viral reference genomes (Supplementary Table S1) using segemehl (52,53) (version 0.3.4) with the ‘-S flag’ to enable split-read alignment mode. The trns.txt output files were used as input to our in-house script plot_heatmaps.py, which selects and counts the intersegmental chimeric reads and generates heatmaps of pairwise segment interactions. Multiple trns.txt files from biological replicates were used as input to generate ‘merged’ interaction heatmaps. We then extracted interaction clusters from these ‘merged’ heatmaps by manual inspection. ‘Merged’ heatmaps and extracted interaction clusters are accessible from the OSF repository. For each interaction cluster, we determined the peak position and selected interaction regions by extending the peak position by 19 nucleotides in each direction. The number of chimeric reads mapping to the resulting 39-by-39 nucleotide window was used to generate a count table of the interaction regions using the in-house script make_counttable.py. We normalized these raw chimeric read counts to the total number of mapped chimeric reads using the in-house script get_library_size.py, resulting in reads per million (RPM) values. For comparative analysis of two different viruses, the raw chimeric read count tables were used as input to a modified DEseq2 (54) script called run_DESeq2.r. Of note, this analysis requires at least two biological replicates and was therefore not performed for samples i-iii shown in Figure 6. To visualize our vRNA interactome data, we used circos (55) (v0.69–8). RPM values are provided in Supplementary Table S2. DEseq2 data are provided in Supplementary Table S3.

### Classification of high- and low-frequency vRNA–vRNA interactions

To categorize the vRNA–vRNA interactions detected in each virus (or condition) into low- and high-frequency contacts, we defined cutoff points using a previously published analytical method (56). Cutoff points are indicated for all samples in Supplementary Figure S2.

### RNA structure prediction and calculation of ΔMFE values

To predict RNA structures and minimum free energy (MFE) values of the 39-by-39 nucleotide interaction regions (described in the previous section), we used RNAcofold from the Vienna RNA package (57) (version 2.5.0) with ‘–noLP -C‘ options and otherwise default parameters. The structure prediction script is available at https://github.com/desiro/Jakob2023_structures. MFE differences (ΔMFE) between wild-type and mutant interactions were calculated as follows: MFE_mut_ − MFE_WT_. MFE and ΔMFE values are provided in Supplementary Table S3.

### Prediction of synonymous mutations

Synonymous mutations aimed at disrupting SPLASH-identified vRNA–vRNA interactions were predicted using the SilentMutations (SIM) tool (58). Parameters were varied to increase the MFE of the targeted interaction by at least 40% whenever possible.

### Next-generation-sequencing (NGS) of virus stocks

To sequence viral stocks, RNA was extracted from cell culture supernatants using the Quick-RNA Viral Kit (Zymo Research; R1034). Ribosomal RNA was depleted with RiboMinus Eukaryote Kit v2 Kit (Thermo Fisher Scientific; A15020). Paired-end libraries were then prepared using the NEBNext Ultra II Directional RNA Library Prep Kit for Illumina (NEB, E7760). A total of 8 pM library was sequenced on an Illumina MiSeq instrument (MiSeq Reagent Kit v2, 300 cycles, Illumina; MS-102-2002). The de-multiplexed raw reads were subjected to a custom Galaxy pipeline. Briefly, the raw reads were pre-processed using fastp (50) (version 0.23.2) with default parameters and mapped to the wild-type SC35M reference genome (Supplementary Table S1) using BWA-MEM (59) (version 0.7.17) with default parameters. Variants (i.e. SNPs and INDELs) were called using the ultrasensitive variant caller LoFreq (60) (version 2.1.3.1), which requires a minimum base quality of 30 and coverage of at least 20-fold. The called variants were then filtered based on a minimum variant frequency of 5% and the strand bias support. Finally, consensus sequences were constructed using bcftools (61) (version 1.9). Regions with low coverage (< 20x) or variant frequencies between 30 and 70% were masked with Ns. An overview of the mutations detected is provided in Supplementary Table S1.

### Statistical analyses

Statistical analyses of PFU titers, relative HAU-to-PFU ratios, and relative vRNA levels were performed using GraphPad Prism 9.4.1, unless otherwise noted. An unpaired t-test was used to compare viral titers. Statistical analysis of relative HAU to PFU ratios and relative vRNA levels was performed using a one-sample t-test. Two-tailed *P* values were corrected by the Bonferroni-Holm method using StatistikGuru (62).Two-tailed *P* values < 0.05 (*), <0.01 (**), and < 0.001 (***) were considered significant. Pearson correlations between the top 30 interactions or the 15 HA segment interactions found in cell-attached, released, and ultracentrifuged virions were performed using GraphPad Prism 9.4.1 with the assumption that the log_2_-transformed (RPM + 1) values were normally distributed.

## RESULTS

### Global detection of vRNA–vRNA interaction network changes using RNAswarm

**Figure 1.**
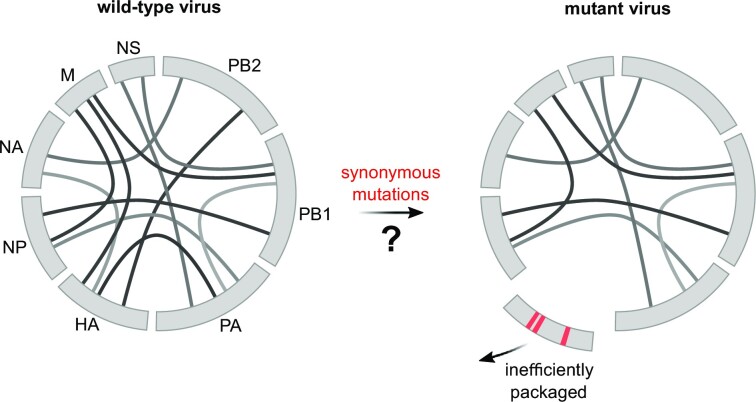
Strategy to identify vRNA–vRNA interactions coordinating IAV genome packaging. To test the role of vRNA–vRNA interactions identified by SPLASH in coordinating IAV genome packaging, we disrupted prominent vRNA–vRNA interactions of the HA segment by introducing synonymous mutations. If the targeted interactions are critical for the genome packaging process, we anticipated reduced packaging of the HA segment.

Similar to the previous identification of TPS by mutational analysis (20,24,26–28,30–34), we hypothesized that disruption of SPLASH-identified interactions by mutagenesis should affect coordinated genome packaging (Figure 1). To test this idea, we needed a SPLASH workflow capable of detecting global changes in the vRNA–vRNA interaction networks of a mutant virus compared to the wild-type virus. Since no such workflow existed, we adopted the original SPLASH workflow (38,45,46) and added RNAswarm, a bioinformatics pipeline that compares the interaction frequencies between different SPLASH-identified networks. We evaluated our new workflow using two chimeric A/PR8 (H1N1) viruses, containing PB1 and NA segments of A/Wyoming (H3N2), as described by Dadonaite *et al.* (38). One of these viruses, rPR8:Wy-PB1/NA, carries wild-type Wyoming PB1 and NA segments, while the other, rPR8:Wy-PB1/NA_Ud-sub_, harbors four nucleotide substitutions in the NA segment derived from A/Udorn/1972 (H3N2). These nucleotide substitutions are critical for forming a vRNA–vRNA interaction with the Wyoming PB1 segment, which is detected by SPLASH and promotes co-packaging of both genome segments (38). By applying our SPLASH workflow to these chimeric viruses, we confirmed this interaction in rPR8:Wy-PB1/NA_Ud-sub_ and its absence in rPR8:Wy-PB1/NA (Supplementary Figure S1A and B). Importantly, RNAswarm detected that this interaction was highly enriched in rPR8:Wy-PB1/NA_Ud-sub_ compared to rPR8:Wy-PB1/NA (Supplementary Figure S1C), consistent with its thermodynamic stabilization due to the introduced mutations (Supplementary Figure S1D). Thus, our new workflow allowed us to (i) identify a previously validated vRNA–vRNA interaction that is critical for IAV genome packaging and (ii) to measure global variations in interaction frequencies between different networks.

### An HA segment lacking high-frequency vRNA–vRNA interactions is properly packaged

**Figure 2.**
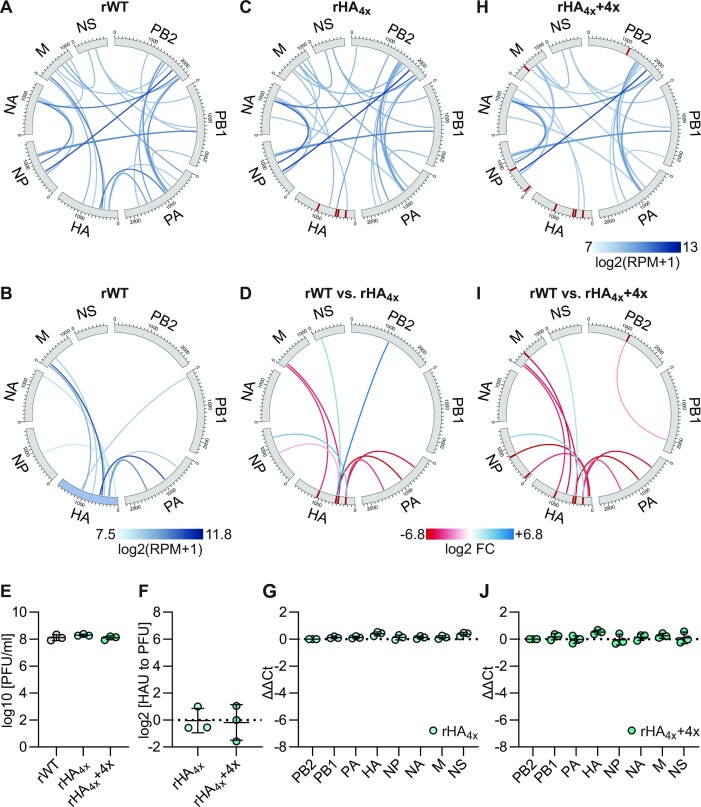
Evaluation of high-frequency vRNA–vRNA interactions detected in SC35M. (**A**, **C** and **H**) Circos plots of the top 30 vRNA–vRNA interactions of (A) rWT, (C) rHA_4x_, and (H) rHA_4x_+ 4x. Shown are log_2_transformed (RPM + 1) means of seven (rWT) or three (rHA_4x_ and rHA_4x_+ 4x) biological replicates. Segments are shown in negative sense from 3′ to 5′. Red bars within the segments indicate the mutated interaction loci. (**B**) Circos plots of the high-frequency interactions involving the HA segment (highlighted in blue) in rWT. (**D**, **I**) Differential vRNA–vRNA interactions detected by DESeq2 analysis between rWT and (D) rHA_4x_ or (I) rHA_4x_+ 4x. Red links indicate interactions lost in mutants compared to rWT and ranked in the top 100 in rWT (log_2_ FC <−1, *P*< 0.01). Blue links indicate interactions gained in mutants compared to rWT and ranked in the top 100 in mutants (log2 FC > 1, *p*< 0.01). (**E, F, G, J**) To measure viral infectivity and segment packaging of the mutant viruses compared to rWT, MDCK-II cells were infected at an MOI of 0.001 PFU per cell. (E) The amount of infectious particles was determined at 24 hpi by plaque assay. The log_10_transformed plaque-forming units per milliliter (PFU/ml) are shown (*n* = 3 for each virus). (F) The relative number of noninfectious particles produced by the mutant virus compared to rWT was determined by hemagglutination assay and plaque assay at 24 hpi. Log_2_transformed fold changes are shown (*n* = 3 for each virus). (G, J) The amount of the eight genome segments packaged into mutant virions relative to wild-type virions was measured by RT-qPCR at 24 hpi. Log_2_transformed fold changes normalized to the amount of PB2 segment are shown (*n* = 3 for each virus).

With this SPLASH workflow in hand, we set out to map vRNA–vRNA interactions for functional evaluation. We performed seven SPLASH replicates on the H7N7 subtype strain SC35M (rWT) and identified more than 1500 vRNA–vRNA interactions that varied in frequency over three orders of magnitude (Supplementary Figure S2 and Supplementary Table S2). Ranking all interactions according to their detected frequencies revealed a biphasic distribution that allowed us to categorize them into low- and high-frequency interactions using a previously published cutoff point identification method (56). Thus, we identified 62 high-frequency vRNA–vRNA interactions (Supplementary Figure S2 and Supplementary Table S2), of which the top 30 ones formed an interconnected network with interaction loci distributed along the entire length of the vRNAs (Figure 2A), similarly to networks previously detected for other IAV strains (37,38).

We hypothesized that removal of multiple vRNA–vRNA interactions from a given genome segment should result in a packaging defect. To test this, we focused on the HA segment which plays an important role in the emergence of pandemic IAVs (63). Within the HA segment we detected ten high-frequency interactions (Figure 2B and Supplementary Figure S3A). We designed synonymous nucleotide substitutions to disrupt the four highest ranked HA-interactions to the PA and M segments and generated the corresponding mutant virus rHA_4x_. SPLASH and RNAswarm analysis revealed an overall similar interaction network compared to rWT (Figure 2C), but also confirmed the loss of the four targeted interactions as well as the loss of one additional high- and one low-frequency interaction involving the mutated HA vRNA loci (Figure 2D and Supplementary Figure S3B). The loss of these interactions was consistent with reduced thermodynamic stability caused by the introduced disruptive mutations (Supplementary Figure S3C). To assess whether coordinated genome packaging in rHA_4x_ was impaired by these interaction losses, we measured infectious and noninfectious viral particles and the amount of the eight vRNAs packaged into virions. Contrary to our hypothesis, rHA_4x_ showed neither reduced viral growth (Figure 2E) nor increased amounts of noninfectious viral particles (Figure 2F) compared to the wild-type virus, and packaged all eight vRNAs efficiently (Figure 2G), indicating that the interaction losses did not affect coordinated genome packaging.

However, the HA segment of rHA_4x_ gained two new high-frequency contacts with the PB2 and NP segments and retained seven additional high-frequency interactions with other genome segments (Figure 2C and D, Supplementary Figure S3A and B). We speculated that these interactions could mediate efficient packaging of the HA vRNA and therefore designed synonymous mutations to disrupt four contacts to the PB2, NP, and M segments in rHA_4x_. To limit the risk of adding unwanted contacts to the HA segment by mutagenesis, we introduced synonymous nucleotide exchanges at the interaction loci of the partner segments. The corresponding mutant virus, rHA_4x_ + 4x, lost a total of eight high-frequency HA segment interactions and retained only four high-frequency interactions of lower rank as determined by SPLASH and RNAswarm analysis (Figure 2H and I, Supplementary Figure S3A and D). These interaction losses were associated with reduced stability of the targeted interactions (Supplementary Figure S3E). However, despite these large interaction losses, rHA_4x_ + 4x did not show impaired viral growth (Figure 2E) or an increased formation of noninfectious virions (Figure 2F) and still packaged all eight vRNAs efficiently (Figure 2J). Thus, an HA segment depleted of multiple high-frequency vRNA–vRNA interactions identified by SPLASH is properly packaged into virions.

Although vRNAs with mutated packaging signals are often inefficiently packaged *per se* (20,24,26–28,30–34), in some cases they are only inefficiently packaged when competing with their cognate wild-type segment for virion incorporation (38,40). We therefore performed a 7 + 2 rescue assay and co-transfected HEK-293T cells with two plasmids, encoding the HA_wt_ and HA_4x_ segments and the seven plasmids encoding the remaining wild-type vRNAs (38,40,64). After viral particle titration, we analyzed the HA segment of ten individual plaques by Sanger sequencing. While four plaques contained the HA_wt_ segment, the other six plaques contained the HA_4x_ segment, indicating that the vRNA–vRNA interaction losses do not affect HA_4x_ vRNA packaging in a competitive context.

**Figure 3.**
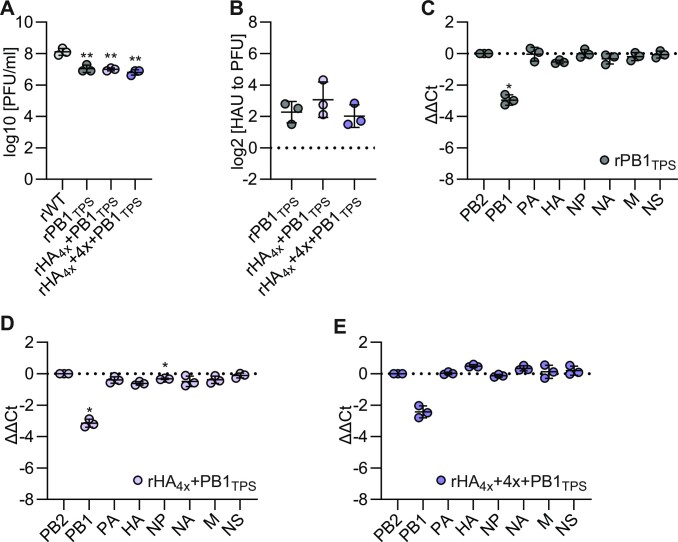
Introduction of a PB1_TPS_ stressor segment into the rHA_4x_ or rHA_4x_+ 4x background does not reveal hidden packaging defects. To measure viral infectivity and segment packaging of the indicated mutant viruses compared to rWT, MDCK-II cells were infected at an MOI of 0.001 PFU per cell. (**A**) The amount of infectious particles was determined at 24 hpi by plaque assay. The log_10_transformed PFU/ml values are shown (*n* = 3 for each virus). (**B**) The relative number of noninfectious particles produced by the mutant virus compared to rWT was determined by hemagglutination assay and plaque assay at 36 hpi. Log_2_transformed fold changes are shown (*n* = 3 for each virus). (**C**–**E**) The amount of the eight genome segments packaged into mutant virions relative to wild-type virions was measured by RT-qPCR at 36 hpi. Log_2_transformed fold changes normalized to the amount of PB2 segment are shown (*n* = 3 for each virus).

We have previously shown that mutation of functional packaging signals may result in ‘hidden’ packaging defects, in which reduced packaging of the mutant vRNA is prevented by redundant packaging signals present in other vRNAs (34). However, a combination of two mutated packaging signals can eventually provoke reduced packaging of both mutant segments. We speculated that the HA segments of rHA_4x_ and rHA_4x_ + 4x might hold such ‘hidden’ packaging defects and would only be inefficiently packaged when combined with a segment harboring a mutated 5′ terminal packaging signal, such as the PB1_TPS_ vRNA described previously (34). To test this scenario, we generated mutant viruses carrying the PB1_TPS_ segment in the rHA_4x_ and rHA_4x_ + 4x backgrounds, designated rHA_4x_ + PB1_TPS_ and rHA_4x_ + 4x + PB1_TPS_, respectively. The rPB1_TPS_ virus carrying only the PB1_TPS_ vRNA showed reduced infectivity compared to rWT (Figure 3A) and an increased production of noninfectious virions (Figure 3B), associated with reduced packaging of the PB1 segment (Figure 3C), as shown previously (34). However, contrary to our hypothesis, the combinatorial mutants rHA_4x_ + PB1_TPS_ and rHA_4x_ + 4x + PB1_TPS_ replicated as efficiently as rPB1_TPS_ (Figure 3A), did not produce more noninfectious viral particles (Figure 3B), and did not inefficiently package any genome segment other than the PB1 vRNA (Figure 3D and E). These results show that an HA vRNA lacking multiple high- and low-frequency vRNA–vRNA interactions detected by SPLASH is efficiently packaged, even in the presence of a vRNA that is known to reveal ‘hidden’ packaging defects.

To exclude that the mutant viruses restored coordinated genome packaging via reversions or compensatory mutations, we performed NGS on viral stocks obtained from rHA_4x_, rHA_4x_ + 4x and rHA_4x_ + 4x + PB1_TPS_ (Supplementary Table S1). All mutant stocks harbored the introduced synonymous nucleotide substitutions. rHA_4x_ did not acquire any unintended mutations. In the rHA_4x_ + 4x + PB1_TPS_ virus stock, we detected a mutation (A609G) in the NA segment that caused an asparagine-to-serine exchange at position 196 (N196S) in the neuraminidase (NA) protein. rHA_4x_ + 4x also contained a mutation (T614C) in the NP segment that resulted in a valine-to-alanine exchange at position 190 (V190A) in NP protein. We did not investigate these mutations further because the NA protein is not known to be involved in IAV genome packaging, and the residue 190 in NP is not exposed on the protein surface, unlike other NP protein residues known to be involved in genome packaging (49,65). Furthermore, none of these mutations was found in rHA_4x_, indicating that they are unlikely to compensate for potential underlying packaging defects in the HA segment. Taken together, these results confirm that the disrupted vRNA–vRNA interactions of the HA segment identified by SPLASH are collectively dispensable for coordinated genome packaging.

### vRNA–vRNA contacts involving TPS are dispensable for coordinated genome packaging

**Figure 4.**
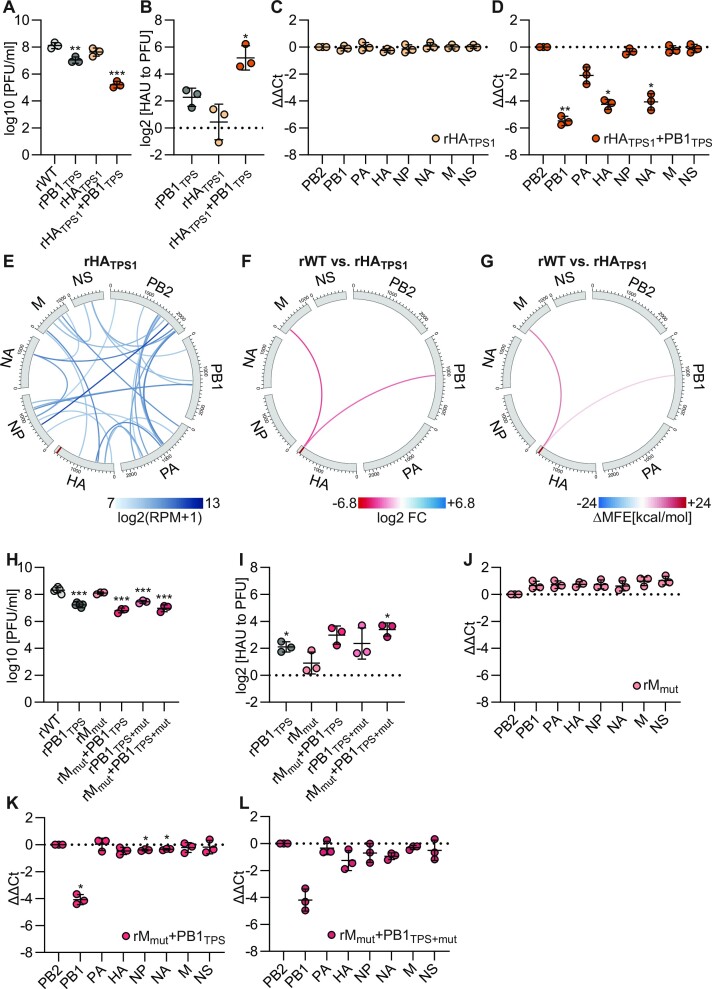
Evaluation of vRNA–vRNA interactions involving the 5′ TPS of the HA segment. (**A**–**D** and **H**–**L**) To measure viral infectivity and segment packaging of the indicated mutant viruses compared to rWT, MDCK-II cells were infected at an MOI of 0.001 PFU per cell. (A, H) The amount of infectious particles was determined at 24 hpi by plaque assay. The log_10_transformed PFU/ml values are shown (*n* = 3–6 for each virus). (B, I) The relative number of noninfectious particles produced by the mutant virus compared to rWT was determined by hemagglutination assay and plaque assay at 24–36 hpi. Log_2_transformed fold changes are shown (*n* = 3 for each virus). (C, D and J-L) The amount of the eight genome segments packaged into mutant virions relative to wild-type virions was measured by RT-qPCR at 24–36 hpi. Log_2_transformed fold changes normalized to the amount of PB2 segment are shown (*n* = 3 for each virus). (**E**) Circos plots of the top 30 vRNA–vRNA interactions of rHA_TPS1_. Shown are log_2_transformed (RPM + 1) means of two biological replicates. The red bar within the HA segment indicates the mutated HA_TPS1_ region. (**F**) Differential vRNA–vRNA interactions detected by DESeq2 analysis between rWT and rHA_TPS1_. Red links indicate interactions lost in rHA_TPS1_ compared to rWT and ranked in the top 100 in rWT (log_2_ FC <−1, *P*< 0.01). (**G**) MFE changes (ΔMFE) of the vRNA–vRNA interactions shown in (F). Red links indicate interactions destabilized in rHA_TPS1_.

Most of the packaging signals identified to date are located at the genome segment termini (18–36). However, it remains to be demonstrated that TPS coordinate IAV genome packaging by forming functional vRNA–vRNA interactions. To test this idea, we screened the HA segment for bona fide TPS and next performed SPLASH analysis to investigate whether the TPS would be involved in coordinating vRNA–vRNA interactions. Previous studies have discovered functional TPS at the 5′ end of the HA segment in different H1N1 viruses (30). Since the 5′ HA vRNA end is not conserved among HA subtypes, we targeted the 5′ end of the SC35M HA segment with three different sets of synonymous nucleotide substitutions between position 1647 and 1713 (3′ to 5′ vRNA), and generated the corresponding viruses, rHA_TPS1_, rHA_TPS2_ and rHA_TPS3_ (Supplementary Table S1). However, none of these mutant viruses showed reduced viral stock titers compared to rWT (Supplementary Figure S4A), suggesting either that the targeted vRNA regions are not critical for coordinated genome packaging or that their functional deficiency is compensated by redundant TPS (34). To distinguish between these possibilities, we generated combinatorial virus mutants carrying the PB1_TPS_ segment in addition to the HA_TPS1_, HA_TPS2_ or HA_TPS3_ segments. Notably, rHA_TPS1_ + PB1_TPS_ showed reduced viral stock titers compared to rPB1_TPS_ (Supplementary Figure S4B), suggesting that the HA_TPS1_ region, comprising nucleotide 1647 to 1677 (3′ to 5′ vRNA), is critical for coordinated genome packaging. We confirmed that viral replication (Figure 4A) and the number of non-infectious virus particles (Figure 4B) of rHA_TPS1_ were comparable to those of the wild-type virus. This mutant also packaged all eight genome segments efficiently (Figure 4C). In contrast, the combinatorial mutant rHA_TPS1_ + PB1_TPS_ was severely attenuated compared to rPB1_TPS_ (Figure 4A) and produced more non-infectious virus particles (Figure 4B). Importantly, rHA_TPS1_ + PB1_TPS_ showed a different packaging defect than rPB1_TPS,_ as it largely failed to package the PA, HA and NA segments in addition to the PB1 segment (Figure 4D). Thus, the HA_TPS1_ region in the 5′-end of the HA segment is a functional packaging signal.

SPLASH analysis revealed that the rHA_TPS1_ virus had an overall similar vRNA–vRNA interaction network compared to the wild-type virus (Figure 4E); however, RNAswarm detected the loss of two low-frequency interactions of the mutated HA_TPS1_ locus with the M and PB1 segments (Figure 4F), possibly due to reduced thermodynamic stability of these interactions in the mutant (Figure 4G). We speculated that one or both of these interactions are critical for coordinated genome packaging and that their loss would cause the packaging defect observed in rHA_TPS1_ + PB1_TPS_. To test this hypothesis, we first introduced disruptive mutations into the M segment interaction loci (rM_mut_). We confirmed the loss of the targeted HA segment interaction in rM_mut_ by SPLASH and RNAswarm analysis (Supplementary Figure 5A–C). Similar to rHA_TPS1_, rM_mut_ replicated to comparable PFU titers as the wild-type virus (Figure 4H), produced similar levels of noninfectious virions (Figure 4I), and efficiently packaged all eight vRNAs (Figure 4J). However, the combinatorial mutant, rM_mut_ + PB1_TPS_, replicated to only slightly lower titers than rPB1_TPS_ (Figure 4H), produced similar levels of noninfectious virions (Figure 4I) and showed a packaging defect only in the PB1 segment but no other vRNAs (Figure 4K). We next tested whether the packaging defect of rHA_TPS1_ + PB1_TPS_ is caused by the combined loss of the vRNA–vRNA interactions of the HA 5′-TPS with the M and PB1 segments. Therefore, we generated rM_mut_ + PB1_TPS+mut_ by introducing synonymous mutations into the PB1 interaction loci which increased the minimum free energy (MFE) of the targeted interaction to the HA 5′-TPS from −28.5 to −9.5 kcal per mol. However, rM_mut_ + PB1_TPS+mut_ did not exhibit a more pronounced growth deficit than rPB1_TPS_ (Figure 4H), did not show increased production of noninfectious particles (Figure 4I), nor did it lose any segment other than the PB1 vRNA (Figure 4L). Collectively, these data suggest that the packaging defect of rHA_TPS1_ + PB1_TPS_ is not due to the loss of the vRNA–vRNA interactions of the HA_TPS1_ region with the M and PB1 segments.

**Figure 5.**
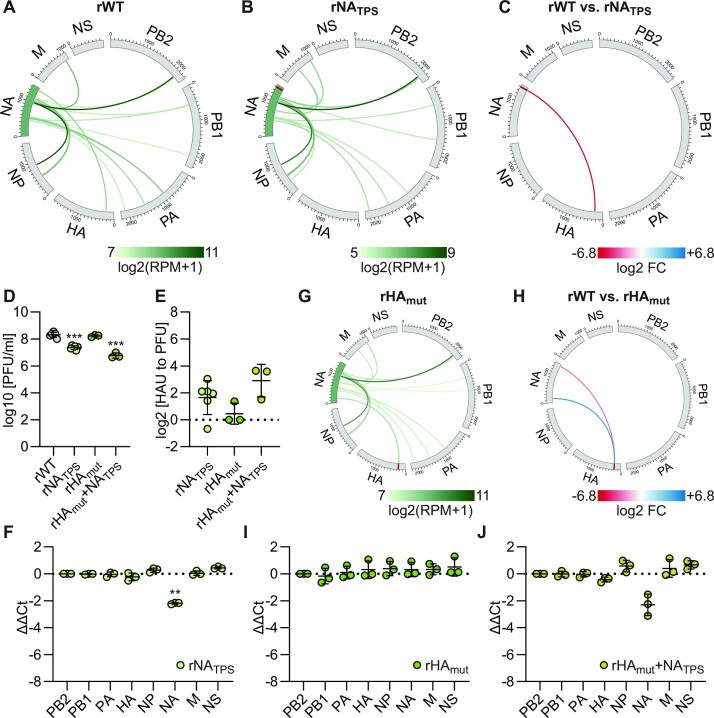
Evaluation of a vRNA–vRNA interaction between the HA segment and the 5’TPS of the NA segment. (**A**, **B**, **G**) Circos plots of the top 15 vRNA–vRNA interactions involving the NA segment (highlighted in green) of (A) rWT, (B) rNA_TPS_, and (G) rHA_mut_. Shown are log_2_transformed (RPM + 1) means of seven (rWT) or two (rNA_TPS_ and rHA_mut_) biological replicates. Red bars within the segments indicate the mutated interaction loci. (**D-F, I, J**) To measure viral infectivity and segment packaging of the indicated mutant viruses compared to rWT, MDCK-II cells were infected at an MOI of 0.001 PFU per cell. (D) The amount of infectious particles was determined at 24 hpi by plaque assay. The log_10_transformed PFU/ml values are shown (*n* = 3–6 for each virus). (E) The relative number of noninfectious particles produced by the mutant virus compared to rWT was determined by hemagglutination assay and plaque assay at 24 hpi. Log_2_transformed fold changes are shown (*n* = 3–6 for each virus). (F, I, J) The amount of the eight genome segments packaged into mutant virions relative to wild-type virions was measured by RT-qPCR at 24 hpi. Log_2_transformed fold changes normalized to the amount of PB2 segment are shown (*n* = 3 for each virus). (**C, H**) Differential vRNA–vRNA interactions detected by DESeq2 analysis between rWT and (C) rNA_TPS_ or (H) rHA_mut_. Red links indicate interactions lost in mutants compared to rWT and ranked in the top 100 in rWT (log_2_ FC <−1, *p*< 0.01). Blue links indicate interactions gained in mutants compared to rWT and ranked in the top 100 in mutants (log_2_ FC > 1, *P*< 0.01).

To extend our evaluation of TPS, we searched the rWT interactome for interactions involving the HA segment and terminal coding regions of other vRNAs. We found a high-frequency interaction of lower rank between the HA segment and the 5′-end of the NA segment (Figures 2B and 5A). To test whether this interaction is critical for coordinated genome packaging, we first targeted the NA segment interaction loci at positions 1375–1405 (3′ to 5′ vRNA) with synonymous mutations and rescued the corresponding mutant virus, rNA_TPS_. SPLASH and RNAswarm analysis confirmed the loss of the targeted interaction (Figure 5B and C) likely caused by the reduced thermodynamic stability of the mutant locus (Supplementary Figure S6A). Viral replication of rNA_TPS_ was impaired compared to rWT (Figure 5D), which was accompanied by an increased production of noninfectious virions (Figure 5E) and impaired packaging of the mutated NA vRNA (Figure 5F). Thus, we identified a functional TPS in the NA segment. To test whether this TPS operates through the SPLASH-identified vRNA–vRNA interaction with the HA segment, we then introduced synonymous mutations into the HA segment interaction locus and rescued the corresponding mutant virus rHA_mut_. SPLASH and RNAswarm analysis confirmed the loss of the targeted interaction (Figure 5G and H), likely due to the reduced thermodynamic stability (Supplementary Figure S6B). Yet, viral replication (Figure 5D), the amount of noninfectious virions produced (Figure 5E), and the packaging efficiency of the eight vRNAs (Figure 5I) in rHA_mut_ were comparable to those of rWT.

However, SPLASH and RNAswarm analysis of rHA_mut_ also revealed one novel high-frequency contact between the NA and HA segments that was not present in rNA_TPS_ or rWT (Figure 5G and H and Supplementary Figure S6B). We hypothesized that this interaction may have compensated for the loss of the interaction involving the 5′-TPS of the NA segment to mediate efficient NA segment packaging in rHA_mut_. If so, this interaction should also restore coordinated genome packaging when grafted onto the rNA_TPS_ virus. To test this, we generated the mutant virus rHA_mut_ + NA_TPS_, carrying the potential compensatory interaction in the background of the lost interaction involving the NA-vRNA 5′-TPS. Contrary to our hypothesis, this mutant virus replicated less efficiently than the wild-type virus (Figure 5D), produced more noninfectious viral particles (Figure 5E), and still packaged the NA segment poorly (Figure 5J), suggesting that the grafting of this vRNA–vRNA interaction into the rNA_TPS_ background cannot correct the packaging defect caused by the mutated 5′-TPS of the NA vRNA. Taken together, these results demonstrate that the packaging defect in rNA_TPS_ is unrelated to the loss of the vRNA–vRNA interaction between the 5′-TPS of the NA segment and the HA vRNA. Thus, the 5′-TPS of the HA and NA vRNAs achieve coordinated genome packaging by means other than the vRNA–vRNA interactions studied.

### Virion release and ultracentrifugation have little effect on SPLASH-identified vRNA–vRNA interaction networks

In the current SPLASH workflow, RNA–RNA cross-linking is performed on digitonin-treated virions that have been released from infected cells and then purified by ultracentrifugation. However, previous electron microscopy and tomography studies suggest that virion release and ultracentrifugation are delicate processes that could potentially affect vRNA–vRNA interactions. For example, it has been speculated that bacilliform viral particles collapse into a spherical shape upon release, which would distort the longer vRNPs (66) and potentially alter their vRNA–vRNA interactions with other vRNPs. Similarly, ultracentrifugation induces shear stress that damages IAV particles (67) and potentially disrupts intersegmental RNA–RNA interactions. In contrast, it has been proposed that budded virions that are still attached to cells contain well-arranged genome complexes with presumably intact vRNA–vRNA contacts (39,41). Therefore, we hypothesized that the vRNA–vRNA interaction networks currently mapped by SPLASH are distorted by the processes of viral release and ultracentrifugation, and that cell-attached virions would be better suited to identify vRNA–vRNA interactions that are critical for IAV genome packaging.

**Figure 6:**
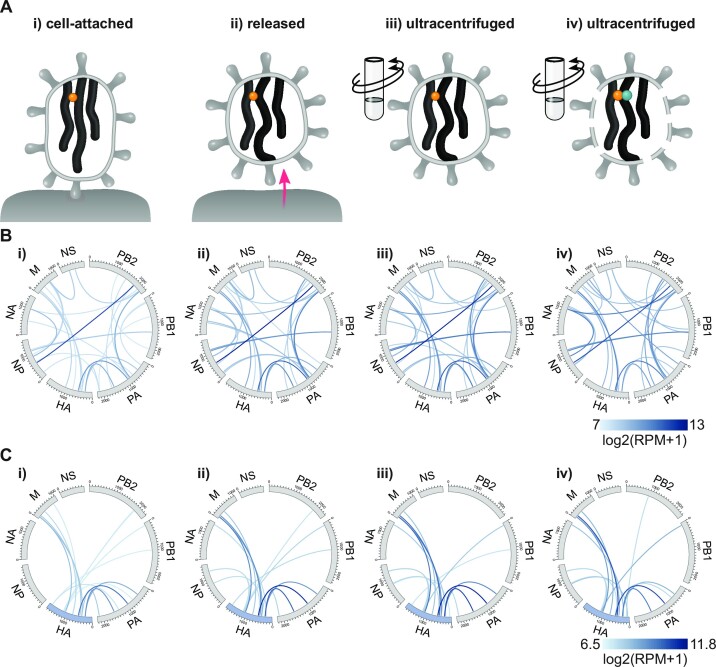
. vRNA–vRNA interaction networks are similar between cell-attached, released and ultracentrifuged virions. (**A**) Schematic representation of the different viral particle populations analyzed. In the CAPTIVE workflow, vRNA–vRNA cross-linking with a clickable psoralen (yellow ball) is performed on either i) cell-attached, ii) released or iii) ultracentrifuged particles. In the original workflow, vRNA–vRNA cross-linking with biotinylated psoralen (green and yellow balls) is performed on iv) ultracentrifuged and digitonin-permeabilized virions. (**B**) Circos plots of the top 30 vRNA–vRNA interactions detected in rWT using the CAPTIVE workflow (i-iii) or the original workflow (iv). Shown are log_2_transformed (RPM + 1) values of one biological replicate for i–iii) and log_2_transformed (RPM + 1) means of seven biological replicates for iv). (**C**) Circos plots of the top 15 vRNA–vRNA interactions involving the HA segment (highlighted in blue).

To test this hypothesis, we developed CAPTIVE (Catching Assembled Particles Via Timely Induced Virus Egress), an experimental approach that allows RNA–RNA cross-linking in specific preparations of either cell-attached, released or ultracentrifuged viral particles (Figure 6A). CAPTIVE consists of three main steps, namely an initial enrichment of newly assembled virions on the surface of infected cells by viral neuraminidase inhibition, followed by a synchronized release of the budded particles induced by an exogenous bacterial sialidase (Supplementary Figure S7A), and a final purification of the released virions by ultracentrifugation. This modular design allowed us to fix vRNA–vRNA interactions in different virion populations, simply by varying the timing of the RNA–RNA cross-linking step. In addition, CAPTIVE is largely compatible with the original SPLASH workflow, requiring only a few adjustments. Specifically, RNA–RNA cross-linking is performed with a clickable psoralen-TEG-azide, which is smaller than the originally used biotin-psoralen and penetrates membranes without digitonin treatment (68). Later in the workflow, a biotin-sDIBO alkyne is added to the psoralen-TEG-azide via click chemistry, allowing enrichment of crosslinked vRNAs on streptavidin beads.

To compare vRNA–vRNA interactions between cell-attached, released and ultracentrifuged virions, we performed CAPTIVE coupled to SPLASH on wild-type SC35M virus. The resulting networks were very similar with the top 30 interactions (Figure 6B) and the top 15 HA segment interactions (Figure 6C) being highly correlated across all three conditions (Pearson correlation coefficients ranging from 0.74 to 0.98) (Supplementary Figure S7B and C). Comparison of the CAPTIVE-derived networks with those obtained with the original workflow (using digitonin treatment and biotinylated psoralen) also revealed high correlation among the top 15 HA segment interactions (Pearson correlation coefficients ranging from 0.61 to 0.87) (Supplementary Figure S7C). Notably, all HA segment interactions mutated in this study were consistently detected at high frequency (Supplementary Figure S7B and C, red dots), and only one novel high-frequency HA segment interaction with the PA segment emerged in the CAPTIVE-derived networks (Supplementary Figure S7B and C, blue dot). Apart from this minor difference, the only major difference was that the CAPTIVE workflow efficiently detected intrasegmental interactions of the 3′ and 5′ vRNA termini (data not shown), suggesting that the psoralen-TEG-azide used in CAPTIVE detects vRNA promoter structures bound by the viral polymerase (17), which are largely missed by the bulkier biotin-psoralen. Taken together, these results demonstrate that virion release and ultracentrifugation have only little effect on the vRNA–vRNA interaction network detected by SPLASH.

## DISCUSSION

The prevailing mechanistic model proposes that the eight IAV genome segments are selectively co-packaged into virions by a network of specific intersegmental RNA–RNA interactions. Recently, extensive and strain-specific vRNA–vRNA interaction networks have been detected in purified viral particles by SPLASH (38). However, whether these networks control genome packaging has not been conclusively demonstrated. In this study, we used mutational analysis to systematically evaluate the relevance of SPLASH-identified vRNA–vRNA interactions for coordinated genome packaging in A/SC35M (H7N7). We found that combined disruption of multiple HA segment interactions does not affect genome packaging. Similarly, we show that interactions involving the TPS of the HA or NA segments are dispensable for coordinated genome packaging. These results suggest that the studied vRNA–vRNA interactions are at most of minor importance for coordinated genome packaging compared to previously described packaging signals.

Since SPLASH was previously used to identify a high-frequency vRNA–vRNA interaction mediating co-packaging of the PB1 and NA segments in H3N2 viruses, our results may seem surprising at first glance. However, it should be noted that the loci involved in this interaction were already suggested by previous mutational mappings (43), which may have facilitated the identification of this particular interaction. In the present study, we mainly evaluated high-frequency interactions of the SC35M HA segment. Although our in-depth mutational analysis did not reveal any novel bona fide vRNA–vRNA interactions involved in genome packaging, it is possible that some of the remaining high- or low frequency interactions are critical for HA segment packaging. These interactions may be thermodynamically stable but poorly cross-linked by psoralen, which is known to preferentially react with interstacked pyrimidines (69). Poor cross-linking of these vRNA–vRNA interactions may result in their artificial underrepresentation in the detected networks, making it difficult to identify them without additional information from mutational mapping.

Alternatively, demonstrating the actual importance of particular vRNA–vRNA interactions for coordinated genome packaging may be complicated by functionally redundant interactions in the network. If two mutually exclusive vRNA–vRNA interactions were able to mediate efficient HA segment packaging, it is possible that disruption of one would be compensated for by the other, resulting in no detectable packaging defect. Thus, despite tremendous interaction losses in our rHA_4x_ and rHA_4x_ + 4x viruses, the remaining vRNA–vRNA interactions might be sufficient to maintain structural integrity of the network.

Although the abundance of detected vRNA–vRNA interactions might indeed reflect to some degree a functional network redundancy, it could also point to systematic limitations in the SPLASH workflow that lead to false-positive interactions. RNA–RNA cross-linking by psoralen is a two-step reaction with a psoralen-RNA monoadduct as an intermediate (70). Like cross-linked RNAs, these monoadducts could be enriched on streptavidin beads and hybridize with free RNAs to form artificial interactions that end up as false-positive chimeric reads in the recovered networks. Although we did not test this possibility here, a previous SPLASH study detected only 4% false-positive chimeric reads (46). Alternatively, many of the vRNA–vRNA interactions detected by SPLASH could be by-products of the genome packaging process. During packaging, the vRNPs are inserted into a narrow viral particle, which may induce vRNP collisions and the formation of a specific set of vRNA–vRNA interactions predetermined by the vRNP arrangement in the (7 + 1) genome complex (41,71,72). Since we detected similar interaction networks between cell-attached, released and ultracentrifuged viral particles, these budding-induced vRNA–vRNA interactions may be robust against further rearrangements. Moreover, such compaction of the viral genome during packaging may disrupt those vRNA–vRNA interactions that are actually critical for viral genome assembly, rendering them largely undetectable within virions. Thus, future studies could examine vRNA–vRNA interactions within infected cells, although this may be challenging if the critical interactions are only transient and restricted to currently poorly defined cellular compartments (73,74).

Finally, it is possible that intersegmental RNA–RNA interactions are not fundamental to IAV genome packaging and are therefore rare and difficult to detect. Indeed, only two functional vRNA–vRNA interactions involved in genome packaging have been identified to date, and these are strain-specific (38,40,43). This continued lack of validated vRNA–vRNA interactions, particularly of those involving the TPS, raises the possibility that other mechanisms control selective vRNP packaging. Previous studies suggest that an interplay between the TPS and viral NP is central to coordinated genome packaging (34). Although speculative, it is possible that the TPS form RNA structures that make specific contacts with another vRNP through intersegmental vRNA-NP interactions.

In conclusion, our study questions the utility of the SPLASH workflow alone for the *de novo* discovery of functional vRNA–vRNA interactions that coordinate IAV genome packaging. We propose that the vRNA–vRNA interaction networks identified by SPLASH are insufficient to explain coordinated vRNP packaging and call for expanded strategies to reveal the ‘true’ genome packaging machinery.

## DATA AVAILABILITY

The data underlying this article are available in the Sequence Read Archive (SRA) at https://www.ncbi.nlm.nih.gov/sra, and can be accessed with the BioProject accession number PRJNA939935.

## Supplementary Material

gkad442_Supplemental_Files
