# Supplementary material for: Sequential disruption of SPLASH-identified vRNA–vRNA interactions challenges their role in influenza A virus genome packaging
Source: Nucleic Acids Res. 2023 May 24;51(12):6479–94. doi: 10.1093/nar/gkad442 (PMC10325904; doi:10.1093/nar/gkad442)
Supplement: gkad442_Supplemental_Files [file gkad442_supplemental_files.zip › Jakob et al. 2023_Supplementary Figures.pdf]

Supplementary data

Sequential disruption of SPLASH-identified vRNA-vRNA interactions challenges their role in influenza A virus genome packaging

Celia Jakob[1,2†], Gabriel Lencioni Lovate[3†], Daniel Desirò[4], Lara Gießler[1], Redmond P. Smyth[5,6], Roland Marquet[7], Kevin Lamkiewicz[3,8,9], Manja Marz[3,8,9,10], Martin Schwemmle[1,2*], Hardin Bolte[1,2*]

Supplementary Figure S1

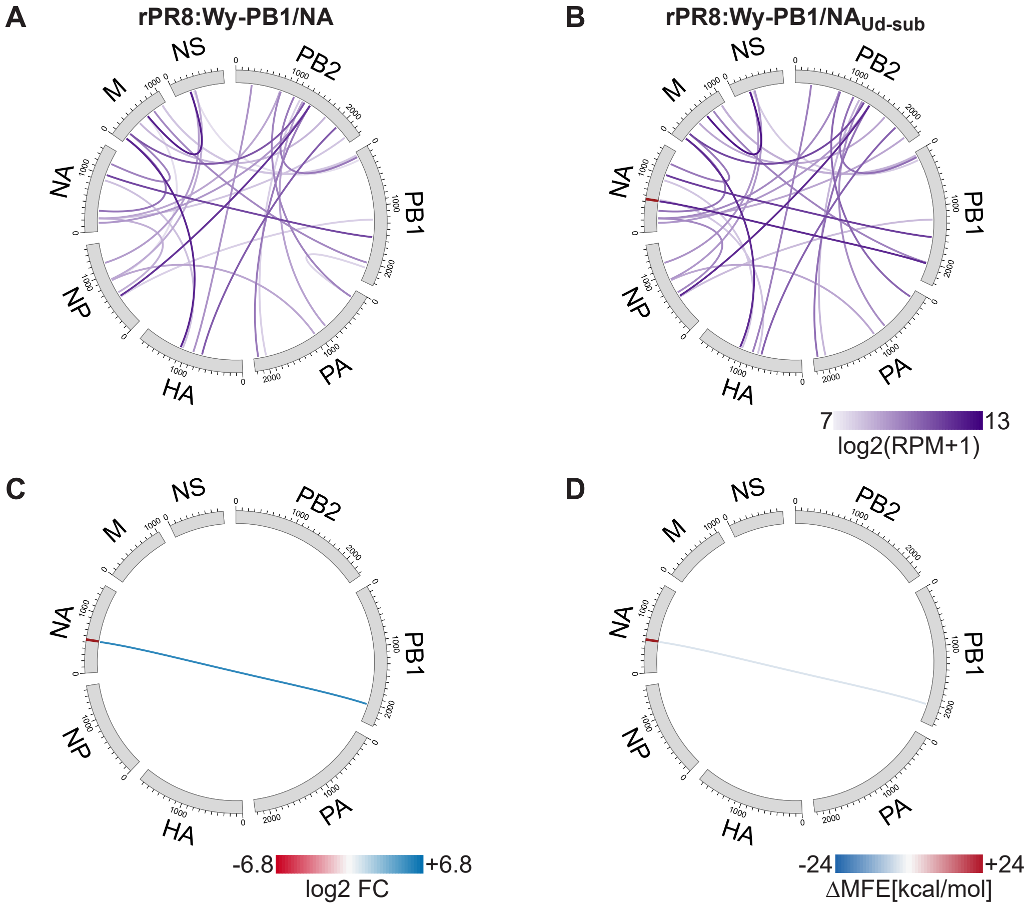

Supplementary Figure S1. SPLASH- and RNAswarm-guided identification of vRNA-vRNA interactions previously described by Dadonaite et al. (**A**, **B**) Circos plots of the top 30 vRNA-vRNA interactions of (A) rPR8:Wy-PB1/NA and (B) rPR8:Wy-PB1/$NA_{Ud\text{-}sub}$. Shown are log_2-transformed means of reads per million +1 (RPM+1) of two biological replicates. Segments are shown in negative sense from 3' to 5'. The Red bar within the NA segment indicates the mutated interaction loci. (**C**) Differential vRNA-vRNA interactions detected by DESeq2 analysis. Blue links indicate interactions gained in rPR8:Wy-PB1/$NA_{Ud\text{-}sub}$ compared to rPR8:Wy-PB1/NA (log2 FC >1, p<0.01). (**D**) Minimum free energy changes (ΔMFE) of the vRNA-vRNA interactions shown in (C). Blue links indicate interactions stabilized in rPR8:Wy-PB1/$NA_{Ud\text{-}sub}$.

Supplementary Figure S2

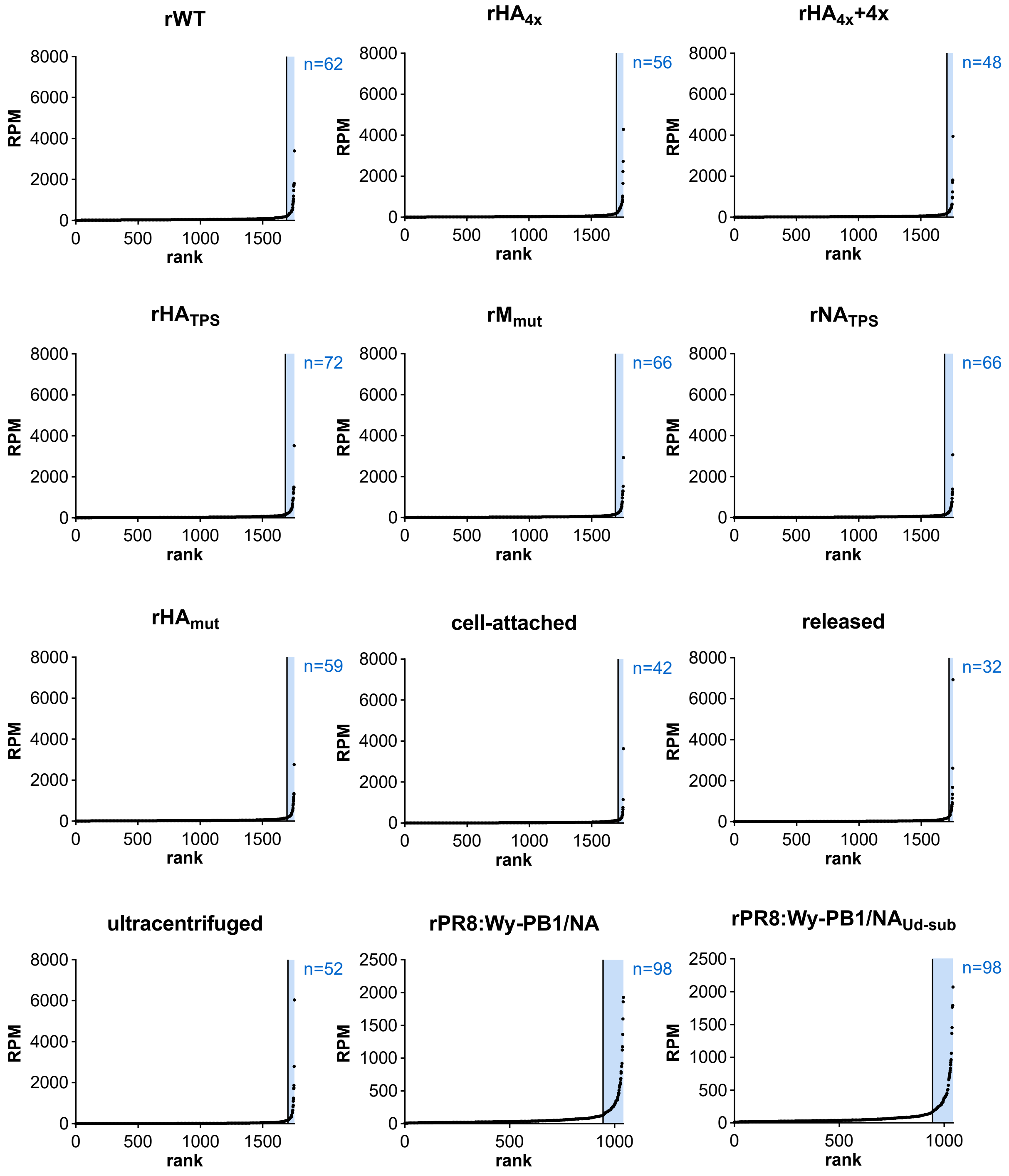

Supplementary Figure S2. Frequency ranking of the vRNA-vRNA interactions detected in this study. All vRNA-vRNA interactions identified in a given virus sample (or condition) were ranked according to their RPM value. Cutoff points for categorizing the interactions into low- and high-frequency interactions were determined for each sample (56). The high-frequency interactions are highlighted in blue and their number (n) is indicated.

Supplementary Figure S3

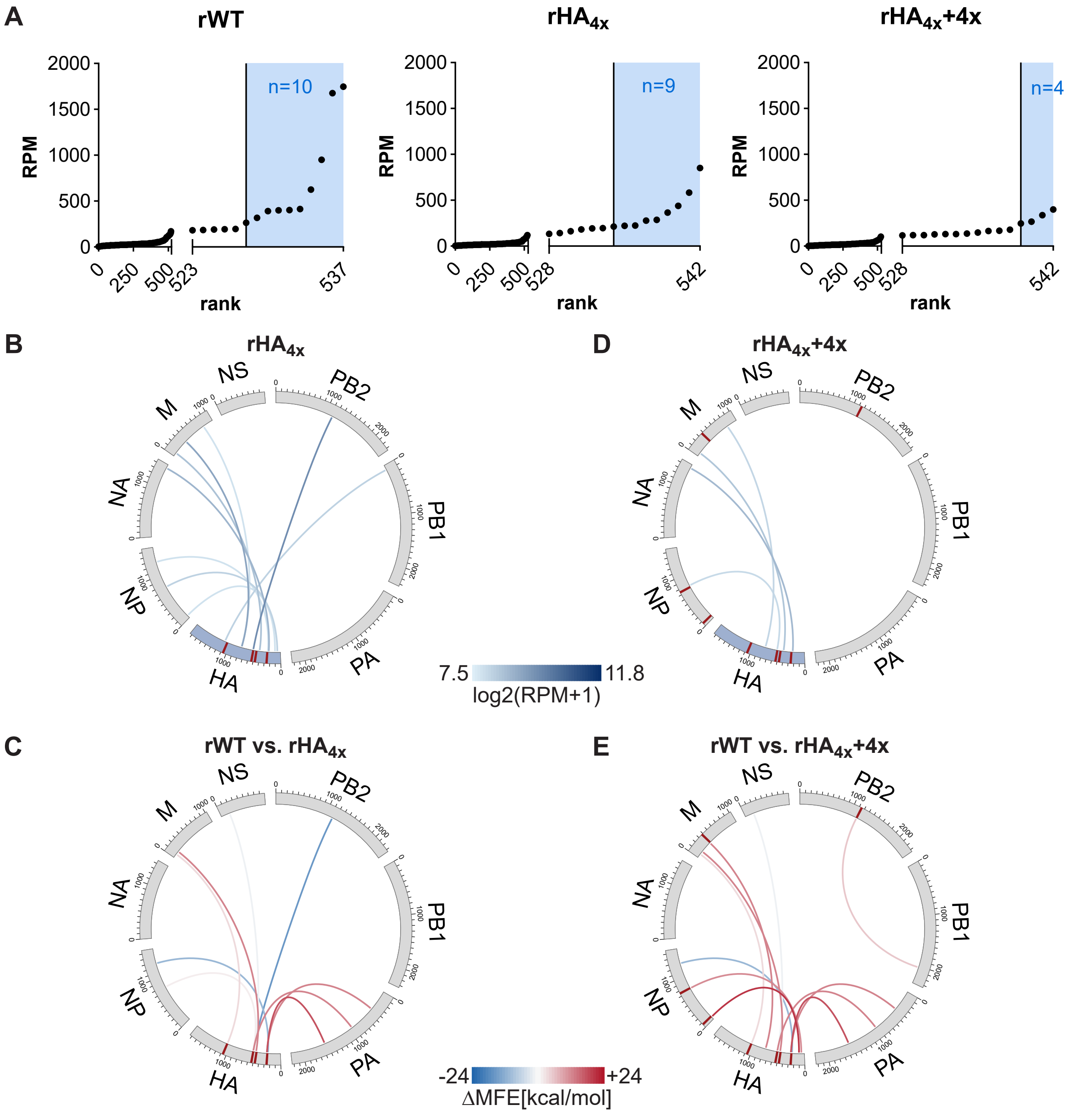

Supplementary Figure S3. High-frequency vRNA-vRNA interactions involving the HA segment detected in rHA$_{4x}$ and rHA$_{4x}$+4x. (**A**) vRNA-vRNA interactions involving the HA segment were ranked according to their RPM value. Cutoff points for categorizing the data into low- and high-frequency interactions were determined for each sample using all identified interactions as shown in Supplementary Figure S2. The high-frequency interactions involving the HA segment are highlighted in blue and their number (n) is indicated. (**B**, **D**) Circos plots of the high-frequency vRNA-vRNA interactions involving the HA segment detected in (B) rHA$_{4x}$ or (D) rHA$_{4x}$+4x. Shown are log_2-transformed (RPM+1) means of three replicates. Red bars within the segments indicate mutated interaction loci. (**C**, **E**) MFE changes (ΔMFE) of the vRNA-vRNA interactions shown in Figure 2D and I. Blue links indicate interactions stabilized in the mutant viruses, while red links indicate destabilized interactions.

Supplementary Figure S4

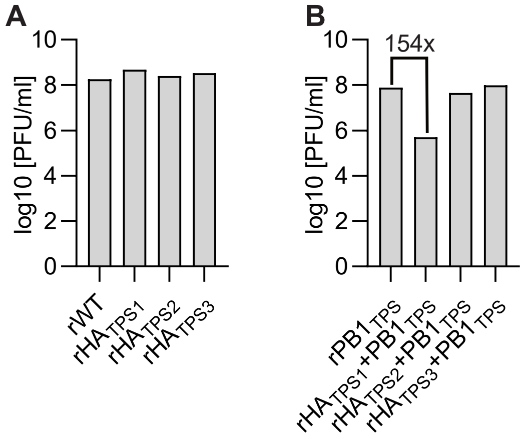

Supplementary Figure S4. Mutational mapping of a terminal packaging signal in the 5'-end of the HA segment. (**A**, **B**) Stock titers of the indicated viruses harboring (A) the wild-type PB1 segment or (B) the $PB1_{TPS}$ stressor segment are shown as log_{10}-transformed PFU/ml (n=1 for each virus). The fold-decrease in the virus stock titer between $rPB1_{TPS}$ and rHA_{TPS1}+$PB1_{TPS}$ is indicated.

Supplementary Figure S5

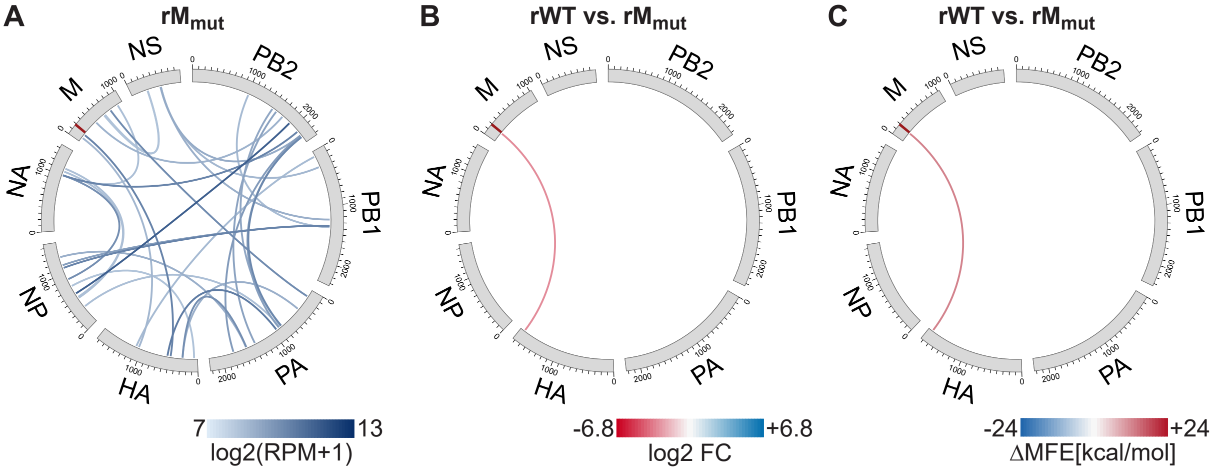

Supplementary Figure S5. Differential vRNA-vRNA interactions detected in rM$_{mut}$. (**A**) Circos plots of the top 30 vRNA-vRNA interactions of rM$_{mut}$. Shown are log_2-transformed (RPM+1) means of two biological replicates. The red bar within the M segment indicates the mutated interaction loci. (**B**) Differential vRNA-vRNA interactions detected by DESeq2 analysis between rWT and rM$_{mut}$. Red links indicate interactions lost in rM$_{mut}$ compared to rWT and ranked in the top 100 in rWT (log2 FC <-1, $p<0.01$). (**C**) MFE changes (ΔMFE) of the vRNA-vRNA interactions shown in (B). Red links indicate interactions destabilized in rM$_{mut}$.

Supplementary Figure S6

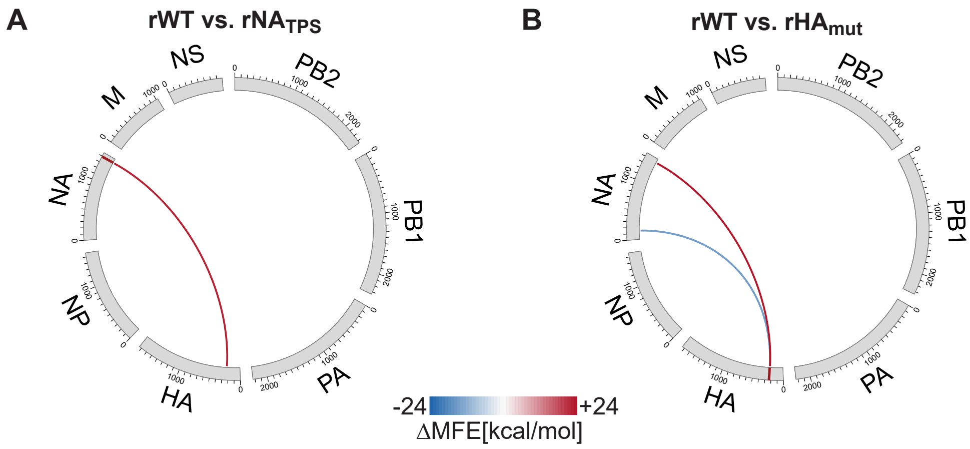

Supplementary Figure S6. MFE changes predicted in rNA$_{TPS}$ and rHA$_{mut}$. (A, B) MFE changes (ΔMFE) of the vRNA-vRNA interactions shown in Figure 5C and H. Blue links indicate interactions stabilized in the mutant viruses, while red links indicate destabilized interactions.

Supplementary Figure S7

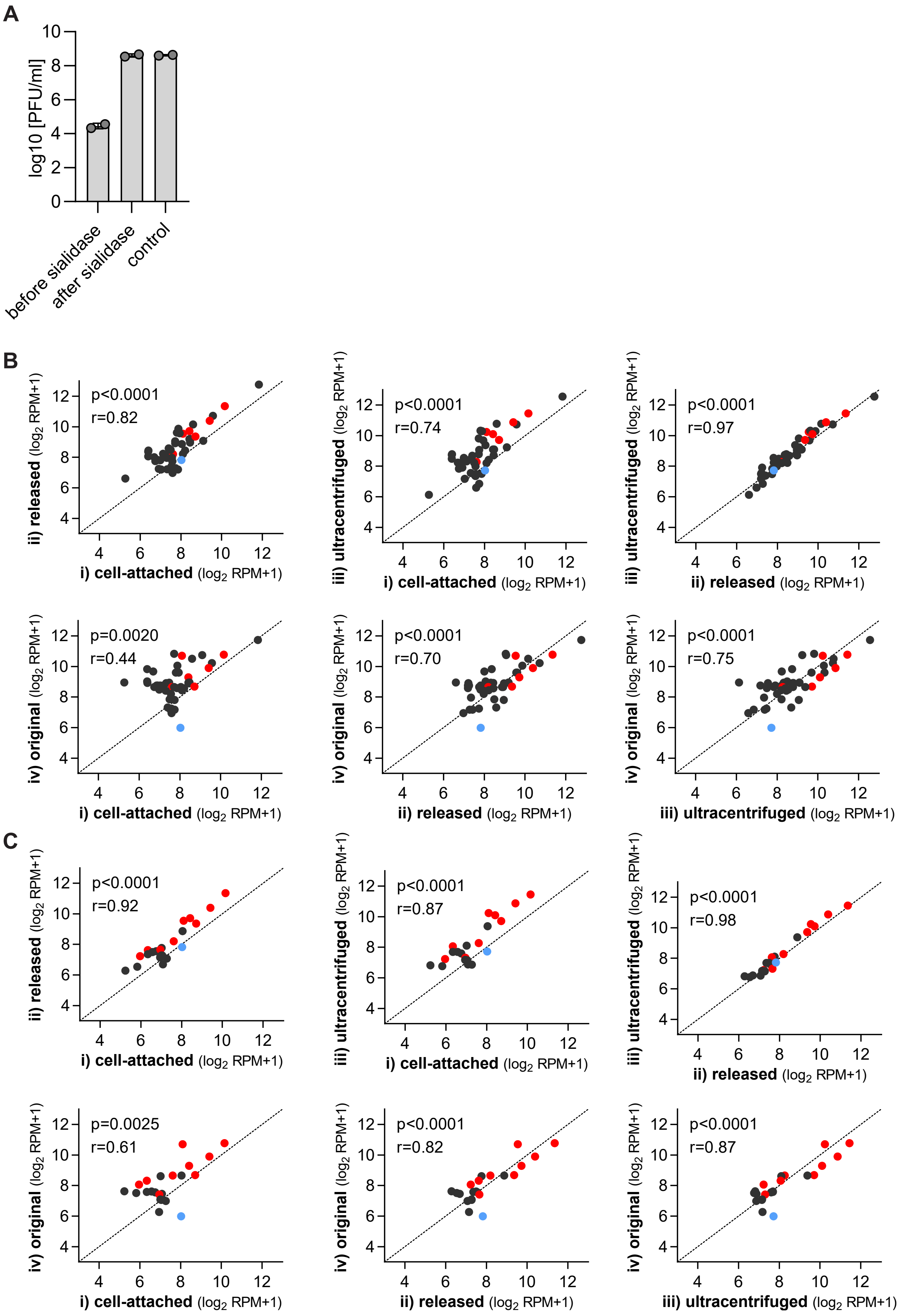

Supplementary Figure S7. Comparative analysis of vRNA interactomes between cell-attached, released and ultracentrifuged virions. (**A**) CAPTIVE allows an enrichment of budded virions on the cell surface and their synchronized release using an exogenous bacterial sialidase. MDCK-II cells were infected at an MOI of 3 PFU per cell and treated with 100 μM of the neuraminidase inhibitor zanamivir to prevent release of newly formed virions. At 13 hpi, cell-attached virions are released by addition of *Vibrio cholera*e sialidase. This procedure allows the recovery of large amounts of viral particles comparable to control infections without neuraminidase and sialidase treatment. The log_{10}-transformed PFU/ml values of two biological replicates are shown. (**B**, **C**) Correlation analysis between the (B) top 30 vRNA-vRNA interactions or (C) top 15 vRNA-vRNA interactions involving the HA segment as depicted in Figure 6B and C. Log_2-transformed (RPM+1) values were used to calculate Pearson correlation coefficients (r). Red dots indicate high-frequency HA segment interactions mutated in this study. The blue dot indicates a high-frequency HA segment interaction that was detected by the CAPTIVE workflow but not by the original workflow.
